# Women bear a burden: gender differences in health of older migrants from Turkey

**DOI:** 10.1007/s10433-020-00596-1

**Published:** 2021-01-26

**Authors:** Verena Krobisch, Pimrapat Gebert, Kübra Gül, Liane Schenk

**Affiliations:** 1 Institute of Medical Sociology and Rehabilitation Science, Charité – Universitätsmedizin Berlin, corporate member of Freie Universität Berlin, Humboldt-Universität zu Berlin, and Berlin Institute of Health, Charitéplatz 1, 10117 Berlin, Germany; 2 Institute of Biometry and Clinical Epidemiology, Charité – Universitätsmedizin Berlin, corporate member of Freie Universität Berlin, Humboldt-Universität zu Berlin, and Berlin Institute of Health, Berlin, Germany; 3 Berlin Institute of Health (BIH), Berlin, Germany

**Keywords:** Ethnicity, Migration, Sex, Elders, Self-reported health, Mixed methods

## Abstract

Studies show that older migrants have poorer health than native populations in Western Europe. To date, little systematic research has explored the differences between men and women within older populations with migration backgrounds. This article examines gender-specific aspects and mediating mechanisms of self-reported health among older migrants from Turkey. Using a mixed method approach, data and results from a quantitative survey and a qualitative study conducted in Berlin, Germany, are analysed and integrated at the interpretive level. Standardised face-to-face interviews were carried out with the help of a network approach with 194 older migrants from Turkey (93 women, 101 men, mean age: 68). Potential mediators showing significant gender differences are included in a parallel multiple mediation analysis. The documentary method is used to analyse 11 semi-structured narrative interviews with first-generation labour migrants from Turkey. Women reported significantly worse subjective health than men (*c* = 0.443, bCI [0.165–0.736]), conveyed through greater functional limitations (*ab* = 0.183, bCI [0.056–0.321]) and emotional loneliness (*ab* = 0.057, bCI [0.008–0.128]). Respondents to the qualitative study perceived that women age earlier and have poorer health due to the burden of performing a greater variety of social roles. Higher levels of emotional loneliness among women could be caused by their experiences of negatively assessed partnerships. Our results show that as a group, older female migrants have an elevated health vulnerability. A broader scientific foundation regarding gender differences in the health of older migrants and their causes is needed to promote gender-sensitive prevention and care for this group.

## Introduction

Since the Second World War and the resulting economic upswing, the subsequent efforts to recruit workers, family reunifications, (post) colonial, and humanitarian migration, Western Europe has become an immigration region. Growing numbers of migrants are now reaching retirement age and entering a phase of life with increased health risks and potential care needs (White 2006).

Studies show that older migrants are in poorer health than their native counterparts (Carnein et al. 2015; Cramm and Niboer 2017; Franse et al. 2018; Milewski and Doblhammer 2015). Even if migrants are in better health initially, evidence suggests that both male and female migrants experience greater deterioration in self-reported health (SRH) during their lives in the host country (Bousmah et al. 2018; Kotwal 2010; Lanari et al. 2015; Reus-Pons et al. 2018). However, little is currently known about how migration and gender are linked in the ageing process – even though gender is a key socio-structural facet of health inequality.

Studies on the health of older migrants often only consider gender as a secondary aspect, descriptive characteristic or control variable. Available findings on gender-specific health aspects indicate that older women with a migration background or belonging to an ethnic minority must be considered a vulnerable group. They show poorer health than their male counterparts in terms of SRH, functional limitations, comorbidities, depressive symptoms, and well-being (Carnein et al. 2015; Cramm and Niboer 2018; Todorova et al. 2013; Yancu 2011).

To our knowledge, there is a significant lack of research on the underlying causes of gender differences in the morbidity of migrants (Gerritsen and Devillé 2009; Llacer et al. 2007; Read and Gorman 2006; Wengler 2011). Broader research on the health of migrants and ethnic minorities from different countries and considerations of various age and origin groups indicates a range of factors potentially relevant to health differentials among older migrant men and women. This includes sociodemographic factors, e.g. age and marital status (Cramm and Niboer 2018; Kotwal 2010; Morawa et al. 2017); socioeconomic factors, e.g. socioeconomic status, income, and working conditions (Cooper 2002; Kotwal 2010; Malmusi et al. 2010; Todorova et al. 2013; Wengler 2011; Yancu 2011); sociocultural factors, e.g. ageing perceptions, coping resources, and loneliness (Carnein et al. 2015; Cramm and Niboer 2018; Todorova et al. 2013; Wengler 2011; Yancu 2011); health-related factors, e.g. health care and health behaviour (Gerritsen and Devillé 2009; Klein and von dem Knesebeck 2018; Read and Smith 2018); as well as migration- and integration-related factors, e.g. length of stay in the host country, language skills, acculturation, and discrimination (Brand et al. 2017; Carnein et al. 2015; Kotwal 2010; Malmusi et al. 2010; Morawa et al. 2017; Todorova et al. 2013).

Therefore, this work aims to shed light on the under-researched issue of gender differences in the health of older migrants and their potential causes. Based on a broad understanding of health that includes mental, sociopsychological, and physical health, we examine differences in SRH and underlying mechanisms among older men and women from Turkey. Migrants from Turkey form a relevant ageing population group in some North-Western European countries including Germany, the Netherlands, and Sweden (Salt 2011; White 2006). With a mixed method approach, we analyse data and triangulate results of a quantitative survey and a qualitative study involving samples of older migrants from Turkey living in Berlin, Germany.

## Methods

### Mixed method approach

Following an exploratory and sequential mixed method design (Fetter et al. 2013), the qualitative study examined concepts of ageing and elderly care in various migrant groups (Schenk et al. 2011). Its findings were used to inform the quantitative cross-sectional study on the care needs of older migrants from Turkey (Krobisch et al. 2014). To explore gender differences in the health of this group, secondary analyses of data from both studies were performed. The results of the secondary analyses and previous qualitative findings were integrated at the interpretive level (Fetter et al. 2013). Thus, qualitatively gained insights in ageing perceptions of first-generation migrants from Turkey served to illuminate the quantitative findings on gender differences in health and its mediating factors.

### Quantitative study

In the quantitative study, data on health status, health behaviour, social well-being, migration, integration, and other sociodemographic and socioeconomic conditions were collected. As a part of the secondary analyses, these data were included in a parallel multiple mediation analysis to examine gender differences in the SRH and potential mediators.

### Data collection and sample selection

Between June and October 2013, in person, standardised interviews were carried out with 194 older migrants from Turkey living in Berlin, Germany. The inclusion criteria for participants were as follows: first, being born in Turkey or the respondents' self-identification as being 'from Turkey', and second, a minimum age of 55, described by participants in the qualitative study as the threshold for being elderly. Participants were recruited via a network approach to reduce migration-related barriers to participation; this approach aims to involve trusted individuals who are anchored in the ethnic community (Yilmaz et al. 2009). Other survey participants were recruited with the help of interviewees who were already part of the survey (snowball sampling) and by interviewers directly approaching people in public places (e.g. men's cafés, parks). Quota sampling was performed using a theory-led approach and included the characteristics of gender, age, and education (see Table 1). An aim was to achieve a balanced number of men and women so that gender-specific analyses could be carried out with a relatively small sample size. The questionnaire was written in German, translated into Turkish, and back-translated for accuracy.

**Table 1:** Sample characteristics in the quantitative study compared with official population data

Characteristics	Sample		Official population data^b^
	*n*	%	%
*Age*^a^			
60–64	75	38.7	33.1
65–69	46	23.7	32.3
70–74	41	21.1	23.4
≥ 75	32	16.5	11.3
Total	194	100	100
*Gender*			
Male	101	52.1	65.9
Female	93	47.9	34.1
Total	194	100	100
*Highest qualification*			
Unskilled/semi-skilled	158	81.4	86.5
Training, apprenticeship, master craftsperson/technician	23	11.9	11.5
University	13	6.7	1.9
Total	194	100	100

^a^Average age of the sample: 68 years; range: 55–88 years; 7 people under the age of 60 were included in the age group 60–64

^b^*Age*: Age distribution of the migrant population from Turkey aged 60 and above, source: data from Germany's 2012 microcensus, own calculation (Statistisches Bundesamt, 2012); no data were available for the age groups 85–90, 90–95, and 95 and above. *Gender:* Gender distribution of the migrant population from Turkey aged 55 and above in the main survey districts of Friedrichshain-Kreuzberg, Mitte, Neukölln and Tempelhof-Schöneberg; source: data from the Statistical Office for Berlin-Brandenburg (2013), own calculation (special data query)*. Highest qualification:* Distribution of the highest professional qualification among Turkish nationals aged 65 and above; source: special analysis of data from the 2002 microcensus (Özcan und Seifert 2006)

### Measures

*Self-reported health (SRH)* was the target variable in this study and measured using a five-point scale (1 = very good, 5 = very bad). In the mediation analysis, SRH was included as a continuous variable (Norman 2010). The analysis considered a variety of mediating variables (see Table 2 for a complete overview of the variables and their categories). *Educational level* was coded following the CASMIN classification (Brauns et al. 2003), in which we included category 1b (general elementary education without vocational qualification) as a part of 'primary education' instead of 'secondary education'. *Subjective income* was measured by asking, 'How often do you have financial worries?'. *German language skills* were assessed subjectively using a five-point scale (1 = very good, 5 = very bad) (Schenk et al. 2006).

*Functional limitations* were included as objective indicators of health, measured in line with SHARE using the six-item activities of daily living (ADL) and the seven-item instrumental activities of daily living (IADL) scales (Mehrbrodt et al. 2017)*. Smoking* as an indicator of risky health behaviour was analysed. *Health behaviour* was surveyed using the items 'I regularly go for walks' and 'I regularly do sports' (yes/no). These two behaviours were combined to create the dichotomous variable of 'physical activity'. If the participant answered 'yes' to at least one of these two questions, the variable 'physical activity' (= yes) was created. The analysis also included the items 'I eat healthily' and 'I have regular medical check-ups' (e.g. cancer screening, blood pressure monitoring, or blood glucose testing).

*Loneliness* as an indicator of social well-being was measured using the De Jong Gierveld and Van Tilburg (2006) six-item loneliness scale. The two subscales reflect emotional loneliness (the lack of an intimate relationship or partner) and social loneliness (the lack of a social network). Each subscale is measured with three items (0 = complete emotional or social embeddedness, 3 = complete emotional or social loneliness). To keep the interviews concise, an item from the geriatric depression scale ('Do you feel that your life is empty?') was used for the emotional loneliness subscale ('I experience a general sense of emptiness'). This had no impact on the evaluation of the scale. Cases with missing entries were excluded from the analysis.

### Analyses

**Fig. 1:**
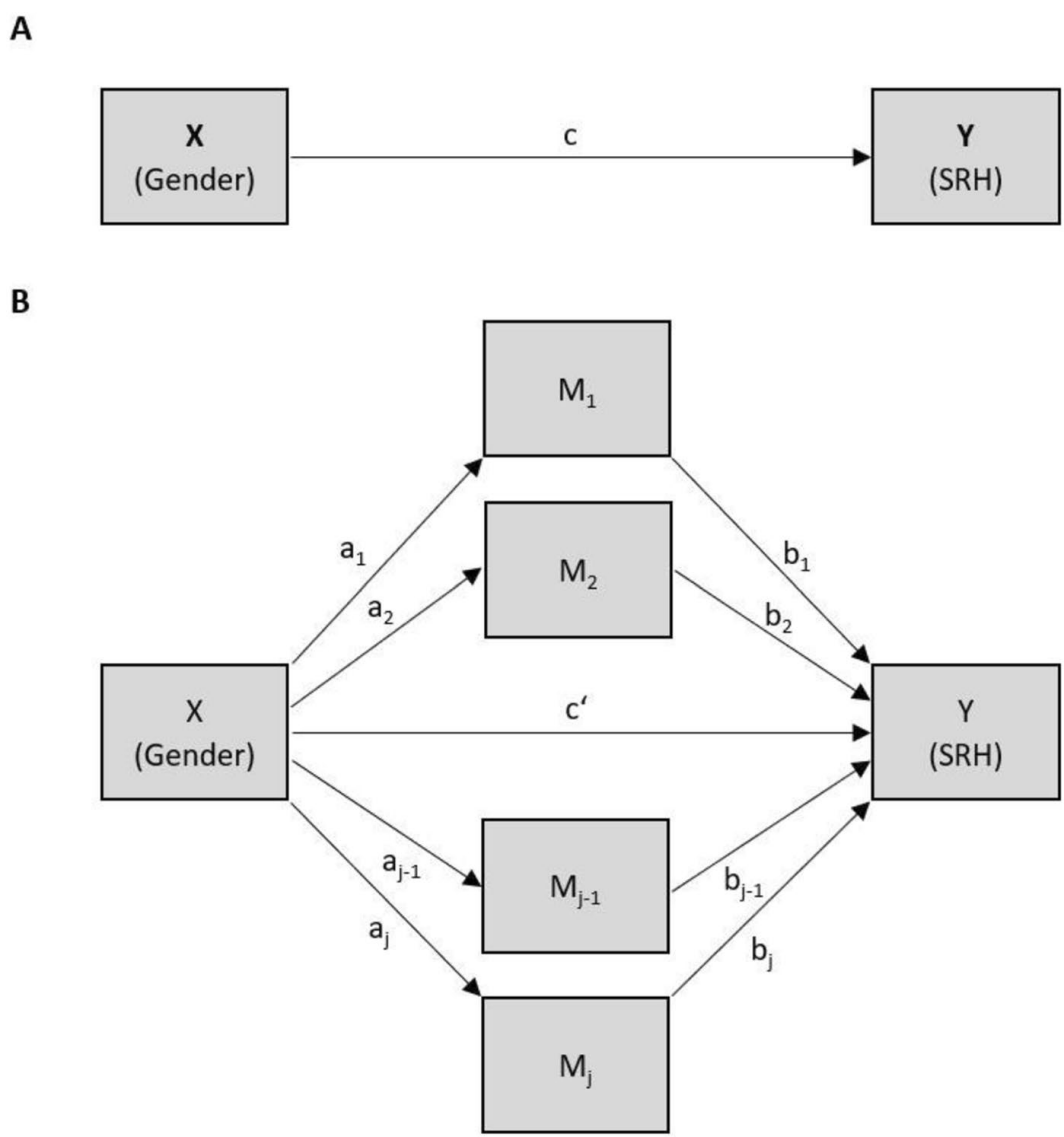
Multiple mediation design with j mediators. (**A**) Gender (*x*) affects SRH (*y*), *c* = total effect. (**B**) Gender (*x*) is hypothesized to indirectly affect SRH (*y*) through *M*_1_, *M*_2_, …, *M*_j_; *a*_1_*b*_1_, *a*_2_*b*_2_, …, *a*_j_*b*_j_ = indirect effects of the mediators, *c*′ = direct effect (Preacher and Hayes 2008, adapted)

The statistical analysis was conducted in two stages. First, descriptive data analysis was performed using Stata IC15 (StataCorp. 2017) to analyse gender differences in SRH and their potential mediators. Frequencies of categorical variables are presented as absolute and relative frequencies. Depending on the distribution, the mean, standard deviation (SD), median, first quartile (Q1), and third quartile (Q3) are considered for continuous variables. Chi-square tests were performed to test gender differences in categorical variables. Independent t-tests or Mann–Whitney U tests were used to analyse gender differences in continuous variables. Second, a parallel multiple mediation analysis explored the relationship between gender and SRH (direct effect) which can be conveyed through other variables (indirect effects of the mediators) (see Fig. 1). By including various potential mediators, it was possible to simultaneously investigate how the mediators are affected by gender (independent variable; 1 = female, 0 = male) and in turn influence the SRH (dependent variable; scores 1 to 5, with 5 being the worst). This method enables testing for the total indirect effect (gender on SRH through all mediators) and specific indirect effects of the mediating factors conditional on the presence of all mediators in the model. Moreover, it reduces the likelihood of parameter bias due to omitted variables (Preacher and Hayes 2008).

The potential mediators were the continuous variables of emotional loneliness and functional limitations (ADL/IADL score). Binary variables were family status, physical activity, and medical check-ups. Multi-category variables were education, smoking behaviour, and ethnic identity. Age was included as a covariate. The mediation analysis was performed using the 'mma' R package (Yu and Li 2017). Mediation effects were estimated based on the unstandardised coefficients in the generalised linear models with the quartile-based bootstrap confidence interval (bCI) based on 1,000 bootstrap samples, and a resample size of 200 was constructed. A significant mediation effect was considered when the bCI did not include 0. The coefficient presents the change rate in SRH (1 = very good to 5 = very bad, with higher values indicating worse SRH) when the variables forming the direct effect or indirect effects change a unit compared to their reference group. Positive β-coefficients indicate the worsening of SRH, such as a positive coefficient in the effect of gender on SRH shows women to have worse SRH than men.

### Qualitative study

Existing results and newly analysed data from the qualitative study focusing on the gender-specific perceptions of ageing among older migrants from Turkey were used to interpret the quantitative results on gender differences in SRH and their potential mediators.

### Data collection and sample selection

A total of 48 migrants in Berlin, Germany were involved in the study, 11 of which were first-generation labour migrants from Turkey whose narratives form the basis for the analysis described below. Semi-structured narrative interviews were carried out between November 2010 and May 2011. Participants were recruited with the help of a network approach similar to the one described in the quantitative methods section. The selection of interviewees was theory-led, based on relevant combinations of sociodemographic characteristics, such as age, sex, and education, as well as characteristics relevant to the specific life context of migrants, such as country of origin and migration context (Kelle and Kluge 1999).

Interviews began with standardised, biography-oriented narrative prompts to elicit narratives of daily life: (1) 'I would like you to start by telling me about your life'; (2) 'And before you came to Germany? Tell me about your life before you came to Germany'; (3) 'And today? Tell me about your life today'. The aim was to shed light on practises that documented non-reflective atheoretical knowledge, unconsciously giving orientation to action (Bohnsack 2008). These prompts were supplemented by the flexible use of questions focusing on the topics of 'age' and 'care'. Interview duration ranged from 30 to 120 min. The average length of an interview was 75 min. At the request of the participants, most interviews were conducted in Turkish.

### Analyses

Qualitative reconstructive research was performed by analysing the recorded, transcribed, and partially translated data using the documentary method (Bohnsack 2008). To begin, we used the 'formulating interpretation' technique to create a topical structure consisting of principal topics and sub topics. In addition, thematically relevant passages from the interviews were summarized and structured to identify the 'immanent meaning'. The 'reflecting interpretation' process then served to explicate orientations and the respondents’ atheoretical knowledge (Bohnsack 2008). In comparative analyses, homologies between passages within an interview and between cases were reconstructed. The interpretation was intersubjectively controlled by four researchers within the research group.

## Results

### Quantitative results

#### Sample characteristics

Table 1 shows the distribution of relevant characteristics in the total sample of 194 older migrants from Turkey. The majority of those in the sample are retired (78.6%) and came to Germany as migrant workers before 1974. The distribution of age, sex, and education in our sample approximately corresponds to official data on the older migrant population from Turkey in Germany.

#### Descriptive results

Table 2 shows gender differences in SRH, age, and the potential mediators. Significant gender differences can be seen in SRH, age, marital status, education, ethnic identity, functional limitations, smoking, physical activity, medical check-ups, and emotional loneliness.

**Table 2:** Descriptive statistics for gender differences in self-reported health, age, and potential mediators

Characteristics	Total	Male	Female	p-value
Self-reported health	*n* = 193	*n* = 100	*n* = 93	0.006
Very good	10 (5.2%)	6 (6.0%)	4 (4.3%)	
Good	38 (19.7%)	27 (27.0%)	11 (11.8%)	
Moderate	89 (46.1%)	46 (46.0%)	43 (46.2%)	
Bad	45 (23.3%)	16 (16.0%)	29 (31.2%)	
Very bad	11 (5.7%)	5 (5.0%)	6 (6.5%)	
*Sociodemographics*				
Age (years)	*n* = 194	*n* = 101	*n* = 93	
Mean (SD)^a^	68 (7)	69 (7)	67 (6)	0.031
Median (Q1, Q3)^b^	66 (62, 72)	68 (63, 74)	65 (62, 71)	
Marital status	*n* = 187	*n* = 100	*n* = 87	0.001
Married/long-term partner	116 (62.0%)	73 (73.0%)	43 (49.4%)	
Widowed/divorced	71 (38.0%)	27 (27.0%)	44 (50.6%)	
*Social status*				
Education	*n* = 194	*n* = 101	*n* = 93	< 0.001
Primary	87 (44.9%)	31 (30.7%)	56 (60.2%)	
Secondary	94 (48.5%)	60 (59.4%)	34 (36.6%)	
Tertiary	13 (6.7%)	10 (9.9%)	3 (3.2%)	
Subjective income	*n* = 184	*n* = 98	*n* = 86	0.792
Always	30 (16.3%)	16 (16.3%)	14 (16.3%)	
Often	28 (15.2%)	13 (13.3%)	15 (17.4%)	
Sometimes	68 (37.0%)	39 (39.8%)	29 (33.7%)	
Never	58 (31.5%)	30 (30.6%)	28 (32.6%)	
*Migration/integration*				
Duration of stay (years)	*n* = 191	*n* = 98	*n* = 93	0.605
Mean (SD)^a^	41 (7)	41 (8)	41 (5)	
Migration pathway (year of immigration)	*n* = 191	*n* = 98	*n* = 93	0.588
Guest worker migration (up to and including 1973)	145 (75.9%)	76 (77.6%)	69 (74.2%)	
Other, e.g. family reunification, humanitarian migration (from 1974)	46 (24.1%)	22 (22.4%)	24 (25.8%)	
German language skills	*n* = 192	*n* = 100	*n* = 92	0.265
Good/very good	34 (17.7%)	22 (22.0%)	12 (13.0%)	
Moderate	92 (47.9%)	45 (45.0%)	47 (51.1%)	
Poor/very poor	66 (34.4%)	33 (33.0%)	33 (35.9%)	
Ethnic identity	*n* = 193	*n* = 101	*n* = 92	0.039
German	11 (5.7%)	7 (6.9%)	4 (4.3%)	
Turkish	110 (57.0%)	63 (62.4%)	47 (51.1%)	
German-Turkish	57 (29.5%)	21 (20.8%)	36 (39.1%)	
Other	15 (7.8%)	10 (9.9%)	5 (5.4%)	
*Physical health*				
Functional limitations	*n* = 190	*n* = 100	*n* = 90	< 0.001^#^
Median (Q1, Q3)^b^	1 (0, 4)	0 (0, 2)	2 (0, 5)	
*Health behaviour*				
Smoking	*n* = 191	*n* = 100	*n* = 91	< 0.001
Never smoked	80 (41.9%)	23 (23.0%)	57 (62.6%)	
Used to smoke	79 (41.4%)	59 (59.0%)	20 (22.0%)	
Currently smoke	32 (16.8%)	18 (18.0%)	14 (15.4%)	
Physical activity	*n* = 192	*n* = 101	*n* = 91	0.009
No	47 (24.5%)	17 (16.8%)	30 (33.0%)	
Yes	145 (75.5%)	84 (83.2%)	61 (67.0%)	
Healthy diet	*n* = 192	*n* = 101	*n* = 91	0.056
No	66 (34.4%)	41 (40.6%)	25 (27.5%)	
Yes	126 (65.6%)	60 (59.4%)	66 (72.5%)	
Medical check-ups	*n* = 192	*n* = 101	*n* = 91	0.009
No	36 (18.8%)	26 (25.7%)	10 (11.0%)	
Yes	156 (81.3%)	75 (74.3%)	81 (89.0%)	
*Social well-being*				
Loneliness scale	*n* = 191	*n* = 101	*n* = 90	0.513^#^
Median (Q1, Q3)^b^	2 (1, 4)	2 (1, 4)	2 (1, 5)	
Social loneliness scale	*n* = 182	*n* = 99	*n* = 83	0.518^#^
Median (Q1, Q3)^b^	1 (0, 3)	1 (0, 2)	1 (0, 3)	
Emotional loneliness scale	*n* = 179	*n* = 97	*n* = 82	0.049^#^
Median (Q1, Q3)^b^	1 (1, 2)	1 (1, 2)	1 (1, 3)	

^a^Standard deviation

^b^(1st quartile, 3rd quartile)

^#^p-value is calculated by the Mann–Whitney U test

#### Mediation analysis

The analysed model indicates that the respondents' SRH is influenced by their gender (*c* = 0.443, bCI [0.165–0.736]) (see Table 3). Although there is no evidence to show that the effect of gender on SRH is mediated by the entire set of potential mediators (_t_*a*_t_*b* = 0.080, bCI [− 0.137 to 0.319]), considering all potential mediators, gender affects SRH through marital status, functional limitations, and emotional loneliness. Women were more likely to be widowed or divorced than the men, which was linked to better SRH (*a*_1_*b*_1_ = − 0.080, bCI [− 0.177 to − 0.017]). Furthermore, women reported greater functional limitations than men. These greater difficulties in performing basic and instrumental everyday tasks resulted in poorer SRH (*a*_2_*b*_2_ = 0.183, bCI [0.056–0.321]). The women also reported higher levels of emotional loneliness than the men and this greater sense of a lack of an intimate relationship is linked to poorer SRH (*a*_3_*b*_3_ = 0.057, bCI [0.008–0.128]). Evidence suggests that gender influences SRH independently of its effect on marital status, functional limitations, and emotional loneliness (*c*' = 0.363, bCI [0.038–0.714]).

**Table 3:** Results of the mediation analysis (*n* = 174)

Self-reported health predicted by	β^a^	bCI^b^
Total effect (c): gender (0 = male^c^, 1 = female)	0.443*	0.165, 0.736
Direct effect (c′): gender	0.363*	0.038, 0.714
Total indirect effect (_t_a_t_b):	0.080	− 0.137, 0.319
*Indirect effects (ab)*		
Marital status (a_1_b_1_) (1 = married^c^, 2 = widowed/divorced)	− 0.080*	− 0.177, − 0.017
Education (1 = primary, 2 = secondary, 3 = tertiary^c^)	0.021	− 0.084, 0.127
Ethnic identity (1 = German^c^, 2 = Turkish, 3 = German-Turkish, 4 = Other)	− 0.005	− 0.073, 0.044
Smoking (1 = never smoked^c^, 2 = used to smoke, 3 = currently smoke)	− 0.062	− 0.189, 0.063
Physical activity (0 = no, 1 = yes^c^)	0.024	− 0.035, 0.094
Medical check-ups (0 = no, 1 = yes^c^)	− 0.012	− 0.065, 0.045
Functional limitations (a_2_b_2_) Score (higher scores = worse)	0.183*	0.056, 0.321
Emotional loneliness scale (a_3_b_3_) Score (higher scores = worse)	0.057*	0.008, 0.128

^a^Unstandardised coefficient of the mediation effect. The range of the SRH is 1 = very good to 5 = very bad, with higher values indicating worse SRH. Positive β-coefficients indicate the worsening of SRH when the effect change a unit compared to the reference group in each effect

^b^Quartile-based bootstrap confidence interval

^c^Reference group

^*^Statistically significant (bCI does not include 0)

### Qualitative results

#### Sample characteristics

Table 4 depicts characteristics of the 11 first-generation migrants from Turkey. The sample shows a balanced gender relationship, an average age of 67.9 years, as well as different marital status and qualification levels. Furthermore, all participants came to Germany as labour migrants during the so-called 'guest worker migration' era.

**Table 4:** Sample characteristics of the qualitative study (*n* = 11)

Characteristics	Years/n
*Age*	
Average	67.9
*Gender*	
Male	6
Female	5
*Marital status*	
Married/long-term partner	6
Widowed/divorced/single	5
*Highest qualification*	
Unskilled/semi-skilled	5
Training, apprenticeship, master craftsperson/technician	4
University	2
*Migration pathway*	
Guest worker migration	11
*Duration of stay*	
Average	42.2

#### Documentary analysis

The results of the qualitative study support the finding that there are gender differences in SRH among older migrants from Turkey. Moreover, the analysis offers possible explanations for the effects of the identified mediators. Although some interviews document the view that men and women do not age differently, other participants showed gender-specific perceptions of ageing. Besides the faster ageing of men, the analysis identified the orientation that women might age earlier and therefore be in poorer health in old age. Both the male and female respondents attributed women's earlier ageing to the associated burdens that result from their voluntary or obligatory assumption of multiple roles in and outside the household.

Hasan (m), a father of five, was born in 1945 and came to Germany as a migrant worker at the beginning of the 1970s. He links the many burdens women bear to the possibility that they age faster than men.

I: Right, Uncle Hasan, do you think women age differently to men? As in, how do women age and how do men age? Are there differences? Or is everyone the same?

H: Of course, there is a difference, but it varies from person to person. Ageing in women, the way women age can be more than in men […] For some people, it's a physical thing. But, of course, men could also be more robust. But when they have led a good life, looked after themselves. Then, she is home with the kids, even in the age her child comes from outside, she has to make them food, she has to give them at least a glass of tea. When a guest comes, the woman has to fight. She does not even leave the kitchen for the guest. Of course, //hmh// especially in our culture it is possible, you are a henpecked husband (laughs) well the man, what has he lost in the kitchen anyways? Because of these words, the man is afraid to go in the kitchen.

Initially, Hasan denies the question that everyone ages the same. He juxtaposes the diversity of individual ageing experiences with a universal, homogeneous ageing. Then, Hasan picks up the category of difference offered by the interviewer in the question and differentiates between men and women. Women age, in his perspective, earlier than men. He speaks of age as a biological matter ('a physical thing') that is also connected to how one acts. If men can maintain a healthy lifestyle, they can protect themselves from ageing. Women, according to Hasan, are not so successful at this. He describes this difference as a result of women's day to day tasks; childcare and the household are women's responsibility. In this, Hasan’s orientation that women age earlier because of the greater burdens associated with fulfilling the duties traditionally assigned to them is shown. Hasan explains the gender-specific division of labour in a change-resistant concept of men ('henpecked husband'). Later in the interview (not quoted), alongside what he sees as a seemingly endless list of female-specific duties (which can also include gainful employment), Hasan reiterates that women may age earlier.

Nihal (f), born in 1944, shares Hasan's orientation. She came to Germany as a worker in 1970, is divorced and has two sons. Like Hasan, Nihal sees women as carrying a heavier burden than men. She seems convinced that the greater diversity of social roles makes women age faster physically.

I: //erm// Okay, do you think women age differently to men?

N: What is that supposed to mean, women and men […] When a woman works. She has a job, and she is also responsible for the child. Dishes, cooking, when guests come to visit […] cleaning windows, ironing, illnesses and everything. Everything is burdened by the woman. How can the woman be in the same situation? I mean, the woman is not a woman – she can't be a woman. It is the life of a slave. Can a woman bear that much? You carry a child in your womb for nine months. Sleep, feeding, diapers, the doctor and goodness knows what else. You are responsible for everything and then you're also supposed to go out and work. I was not human when I went to work. My husband would leave the house in the morning and no one knew when he'd be back that evening. He also did not pay any attention to the children.

Nihal stresses the great burden women shoulder, the responsibilities that traditionally fall to their gender without spousal support compounded by gainful employment. Nihal's use of the word 'slave' in contrast with 'woman' and 'human' suggests that she feels women do not have autonomy. Without any time for herself, Nihal lacks an important source of regeneration. By saying that she could not rely on her husband coming home in the evening, Nihal characterises her husband's presence in the family by a lack of commitment.

The experience of being unable to rely on a partner for support is also documented in the narration of another female respondent. Leyla, a twice-divorced mother of two, reflects on the behaviour of men towards their wives. She was born in 1936, moved to Germany as a migrant worker in 1974, and lives in a nursing home. Her experiences have given her a negative image of men.

L: Even if I got married […] and then became […] ill, I'd like to see whether my husband would look after me. Lots of men leave their wives. He takes her away and hands her over to the house of worship or a care home somewhere. Done and dusted!

Leyla feels that men behave irresponsibly towards their wives by leaving them or institutionalising them when they are in times of need. When talking about women being reliant on men, she implicitly expresses the view that women might age faster or need care earlier than men.

## Discussion

### Main findings and triangulation

The descriptive and mediation analysis showed that the older women from Turkey had significantly poorer SRH than the men. According to our mediation model, this gender difference is conveyed through higher levels of functional limitations and emotional loneliness among the women. The marital status 'widowed/divorced', which was more frequent among women, had a positive effect on health. However, this effect appeared to be offset by the two negative mediation effects, functional limitations, and emotional loneliness. There is also evidence that gender influences SRH independently from the identified mediators. The qualitative findings on perceptions of ageing among first-generation migrants from Turkey suggest that among other orientations, respondents perceive women as ageing earlier and being in poorer health. Both male and female respondents said that this faster ageing could be caused by women having to play a greater variety of roles. They felt that women had to withstand a heavier burden than men because they go out to work while also shouldering most of the responsibility for the household and the children. The higher levels of emotional loneliness could be related to the women's negative experiences with insufficiently supportive husbands.

### Discussion of main findings

Our finding that older female migrants from Turkey have significantly worse SRH than the male migrants corresponds to gender differences found in other studies. In a study of migrants with Turkish nationality in Germany, Wengler (2011) showed that first-generation female migrants rated their health significantly lower than their male counterparts. In the Netherlands, Gerritsen and Devillé (2009) investigated gender differences in the health of various ethnic minorities. They found the largest differences in the group of Turkish migrants, which also reflected worse SRH among the women. Although both studies used a younger population than the present study, evidence for the loss or even reversal of health benefits among migrants over time in their host country (Bousmah et al. 2018; Kotwal 2010) suggests that a higher burden of disease will occur with increased age. Similarly, findings from Carnein et al. (2015) show that older female migrants from Turkey (aged 50–79) in Germany spend more years with functional limitations than their male counterparts. Comparable gender-related patterns in SRH (and other health outcomes) can also be found in other migrant groups and ethnic minorities. These patterns are reported in studies of older Puerto Rican women (aged 45–75) in the greater Boston area in the United States (Todorova et al. 2013), women from Morocco and Suriname (aged 18 and above) in the Netherlands (Gerritsen and Devillé 2009), and Black Caribbean and Indian women (aged 16 and above) in England (Cooper 2002). However, the literature also suggests the need for a differentiated view of migrant populations and ethnic minorities in terms of gender-related aspects of health. Studies from a variety of countries have found that some groups and health indicators show no significant gender differences in SRH, chronic conditions, and risk of depression (Cooper 2002; Gerritsen and Devillé 2009; Read and Gorman 2006; Yancu 2011). Our finding supplements current research on gender differences in the health of migrants—which largely focuses on younger population groups—with evidence showing that women in older migrant populations are at a health disadvantage. In doing so, it supports existing findings that point to the particular vulnerability of older women from Turkey living in Europe (Kotwal 2010; Verest et al. 2019).

Moreover, our study contributes an analysis of possible causes of gender differences in SRH among older migrants illuminated by the interpretive integration of the quantitative and qualitative results. Accordingly, the poorer subjective health of the women is mediated by their worse objective health (functional limitations) and this might be partially caused by the physical burden of fulfilling a greater number of roles. This corresponds with findings from previous studies that indicate poorer objective health (e.g. in terms of functional limitations, acute, and chronic conditions) among women from Turkey of various ages compared to their male counterparts (Carnein et al. 2015; Gerritsen and Devillé 2009; Morawa et al. 2017). In addition, Todorova et al. (2013) found a negative association between the objective health (number of medical conditions, functional problems) and SRH of older Puerto Ricans in the greater Boston area. The multiple roles performed by first-generation female migrants from Turkey appear to result in cumulative health effects from the combination of traditional gender roles with the labour migrant lifestyle in Germany. As per their gender-specific socialisation from the country of origin (Diehl et al. 2009; Kretschmer 2018), women continue to be responsible for the household and childcare, while also holding down gainful employment in the Western European economic system. However, the multiple burdens could also reflect a gender-specific asymmetry in the social system of the country of destination. Gender researcher Regina Becker-Schmidt (1987) terms this the 'double socialisation of women': While men are commonly freed from domestic and family duties, womanhood is socially assigned dual roles, domestic and family work as well as gainful employment.

The mediating effect of emotional loneliness aligns with existing research. Loneliness among older migrants, particularly from Turkey, in various European countries is more prevalent than among the native populations (Fokkema and Naderi 2013; ten Kate et al. 2020, van Tilburg and Fokkema 2020) and a negative factor for their health (Carnein et al. 2015). Todorova et al. (2013) found that low emotional support had a negative impact on the SRH of older Puerto Ricans in the greater Boston area, which indicates a relevant association between emotional loneliness and SRH in migrants. Moreover, our qualitative finding that a (perceived) unreliable relationship might be partly responsible for higher levels of emotional loneliness among women is supported by findings from the Netherlands and Germany. Accordingly, the presence of a partner in the group of adult migrants from Turkey is considerably less protective against emotional and social loneliness than among Dutch adults (Visser and el Fakiri 2016). In groups of older migrants from Turkey and native Germans, a partner only prevented loneliness if the relationship was perceived as good (Fokkema and Naderi 2013). However, the smaller protective effect of a partner could also be related to other factors, such as an emphasis on family and community (Visser and el Fakiri 2016).

The positive effect on SRH of being widowed or divorced that we found among the older female migrants in our study contradicts findings that show, for instance, a significant negative association between the marital status 'divorced/single/widowed' and the well-being of older community-dwelling Turkish people residing in Rotterdam (Cramm and Niboer 2018). However, the gender-specific experience of a lack of a quality relationship in marriage and the multiple roles that women are required to perform could certainly explain the positive mediating effect of this particular marital status.

### Limitations

Given that both of our studies were conducted in Berlin, Germany, and that the quantitative study sample was relatively small and non-probabilistic, the results cannot readily be transferred to the older population from Turkey in Germany and Europe. However, the sample approximately represents the distribution of age and education in official statistics from Germany. Quota sampling, which did not include health and integration characteristics, may be another limitation. Thus, less healthy and more integrated persons who tend to show greater willingness to participate in health studies could be overrepresented in the sample. Another limitation is the lack of comparison groups, e.g. from the German non-migrant population that could provide information about the specificity of the quantitative results for the older migrants. Women might also tend to assess their health more negatively than men due to differences in how they perceive their bodies and the attention they place on their symptoms. Nevertheless, our results show that poorer SRH corresponds with poorer objective health among women. Furthermore, despite translating and back-translating the questionnaire to identify possible errors, mistranslations could have been overlooked.

### Implications

Our results show that older female migrants from Turkey have an elevated health vulnerability. Future research using population-representative data and qualitative methods are needed to shed further light on gender-specific aspects of health and their causes in this migrant group. Comparisons with other older migrants, ethnic minorities, and native population groups should be made, e.g. with non-migrant women of the same age and with similar roles in life. Researchers should consider the qualitative evidence for how gender-specific allocation of roles and emotional loneliness affect the health of older migrants—as well as other socioeconomic, sociocultural, and migration- and health-related explanations. It would also be interesting to investigate whether changes in the gender effect can be seen over time, such as between generations of migrants or during different historical eras of migration. Finally, policymakers and healthcare practitioners should enhance gender-sensitive approaches to health promotion, prevention, and care for older female migrants from Turkey.

## Conclusion

This analysis is one of the first to systematically examine gender differences and their causes in the health of older migrants from Turkey. In addition to further evidence of health disadvantages of older migrant women in Western societies, the paper adds previously lacking explanations showing that reduced objective health and greater loneliness seem to imply poorer SRH among women. Notably, this may be related to their greater burdens due to multiple social roles and feelings of being unsupported in their partnerships.

## Data Availability

Restrictions apply to the availability of these data, which were used under license for this study. Data are available from the authors with the permission of the Centre for Quality in Care (ZQP), Germany.
